# Emotional Speech Markers of Psychiatric Disturbance in Huntington’s Disease

**DOI:** 10.1192/j.eurpsy.2026.12064

**Published:** 2026-07-31

**Authors:** L. Chenain, A. Fabre, H. Titeux, G. Morgado, K. Youssov, C. Clavel, A.-C. Bachoud-Lévi

**Affiliations:** 1 Département d’Etudes Cognitives, École Normale Supérieure, Paris; 2 Faculté de Santé, Université Paris Est Créteil, INSERM U955, Institut Mondor de Recherche Biomédicale, Créteil; 3 INRIA; 4 Faculté de Santé, Université Paris Est Créteil, INSERM U955, Institut Mondor de Recherche Biomédicale, Paris; 5 Service de Neurologie, AP-HP, Hopital Henri Mondor-Albert Chenevier; 6 Centre d’Investigation Clinique, INSERM, Créteil, France

## Abstract

**Introduction:**

Psychiatric symptoms and difficulties in emotional expression are a significant aspect of Huntington’s Disease (HD). Both may be associated or interact, but these associations remain unexplored. To improve patient follow-up, we investigated how emotional expression related to psychiatric symptoms in HD. Speech provides acoustic markers of emotional and psychiatric states across diverse populations, and is readily deployable for digital monitoring to support between-visit follow-up and enhance patient care.

**Objectives:**

To this aim, we developed emoHD, the first HD emotional/psychiatric speech corpus, to investigate the link between emotional expression and psychiatric symptoms.

**Methods:**

We included 102 HD gene carriers and 35 healthy controls (HC) from the REPAIR and BIOHD cohorts. Psychiatric symptoms were assessed using the Problem Behaviors Assessment (PBA), assessing depression, irritability/aggressivity, apathy and obsessive/compulsive scores. Participants performed emotional and non-emotional speech tasks. Their recordings were annotated using three emotional descriptors: primary emotions for a basic emotional characterization; affective phenomena selected based on PBA statements to capture complex HD-associated emotional states; and activation for emotional intensity. We analyzed (1) the emotional descriptors’ relationships using Fisher’s tests, (2) emotional expression differences between HD and HC through permuted t-tests, and (3) the associations between emotions and psychiatric symptoms though stepwise multilinear regressions, using the proportions of the emotional labels as predictors and adjusting for sex, age and clinical group status (HD Integrated Staging System 0-1, 2, 3, HC).

**Results:**

HD patients showed reduced emotional expressiveness compared to HC, with higher neutral activation levels. Affective phenomena were more powerful in identifying emotional expression differences between HD and HC, with HD expressing more *apathetic, confused, depressed, disoriented, frustrated and pessimistic* speech*.* In contrast, they expressed less of the primary emotion *anger,* and the affective phenomena *irritable* and *other.* Expression of specific emotions was congruent with psychiatric symptoms (Table 1).

**Table 1.** Positive associations with the PBA Table 1. Long description.

	Primary emotions	Affective phenomena
Depression		anxious nervous
Apathy	neutral	pessimistic depressed apathetic hopeless
Irritability/aggressivity	sad	quick tempered frustrated
Obsessive/compulsive		fearful apathetic

**Conclusions:**

Emotional speech is a promising marker for psychiatric disturbances in HD. This encourages future work to develop speech-based digital monitoring tools like speech emotion recognition models. This paves the way for a scalable, non-invasive remote assessment of emotional and psychiatric symptoms in HD, with personalized care strategies and timely intervention.

**Disclosure of Interest:**

None Declared

## Long descriptions

### Table 1. Long description

The table consists of three columns: an unlabeled first column for psychological states, Primary emotions, and Affective phenomena.

* Row 1: Depression is associated with the affective phenomena of anxious and nervous. The primary emotions cell is empty.

* Row 2: Apathy is associated with the primary emotion of neutral and the affective phenomena of pessimistic, depressed, apathetic, and hopeless.

* Row 3: Irritability/aggressivity is associated with the primary emotion of sad and the affective phenomena of quick tempered and frustrated.

* Row 4: Obsessive/compulsive is associated with the affective phenomena of fearful and apathetic. The primary emotions cell is empty.

Navigate back to Table 1..

